# Lung cancer-associated membranous nephropathy with positive anti-PLA2R autoantibodies: a case report

**DOI:** 10.11604/pamj.2026.53.119.44958

**Published:** 2026-03-10

**Authors:** Mouna Jerbi, Mouna Riguen, Asma Bettaieb, Mariem Khadhar, Hanen Gaied, Raja Aoudia, Sarra Hadded, Rym Goucha

**Affiliations:** 1 Laboratory of Kidney Pathology LR01SP01, University Tunis El Manar, Tunis, Tunisia,; 2 Nephrology Department, Faculty of Medicine of Tunis, University Hospital Center Mongi Slim, Tunis, Tunisia

**Keywords:** Membranous nephropathy, anti-PLA2R autoantibodies, lung cancer, case report

## Abstract

Membranous nephropathy (MN) is a common cause of idiopathic nephrotic syndrome in adults. The identification of the phospholipase A2 receptor 1 (PLA2R) as a podocyte antigen in adult patients with MN allows clinicians to quickly and accurately diagnose primary MN. Secondary forms associated with malignancy, medications, infection, or autoimmune disease do not generally express anti-PLA2R autoantibodies. We describe a case of a 66-year-old woman who presented with impure nephrotic syndrome, and a renal biopsy showed MN. The diagnosis of secondary MN was initially retained despite positive Anti- PLA2R in light of the discovery of a pulmonary adenocarcinoma. However, nephrotic syndrome persisted after surgical treatment and tumor remission. Remission was only achieved after the use of immunosuppressive therapy. Our case teaches us that even in the presence of an evident etiology for MN, the diagnosis of primary form should always be reconsidered in the absence of remission, especially when anti-PLA2R antibodies are positive.

## Introduction

Membranous nephropathy (MN) is a common cause of idiopathic nephrotic syndrome in adults. Membranous nephropathy is an autoimmune disease caused by antibodies directed to podocyte antigens, which play a main role in controlling the glomerular permeability to proteins [1]. The identification of the phospholipase A2 receptor 1 (PLA2R) and thrombospondin type-1 domain-containing protein 7A (THSD7A) as podocyte antigens in adult patients with membranous nephropathy has strongly impacted both experimental and clinical research on this disease [1]. In fact, it allows clinicians to quickly and accurately, with a specificity approaching 100%, diagnose primary MN. It is classified as secondary when associated with malignancy, medications, infection, or autoimmune disease [1]. The presence of a secondary cause should in no case definitively rule out the primitive form, especially in the presence of anti-PLA2R autoantibodies. Generally, it is the course of evolution that corrects the diagnosis. The MN sometimes represents a therapeutic challenge, especially when it is difficult to distinguish between the primary form and the secondary form. Therefore, this study aimed to report a case of biopsy-proven MN with positive anti-PLA2R autoantibodies, in whom invasive adenocarcinoma of the lung was discovered. The secondary form was initially considered, but the diagnosis was revised in light of the lack of remission after surgical treatment.

## Patient and observation

**Patient information:** a 66-year-old woman, a non-smoker and non-alcoholic, had a history of treated lymph node tuberculosis 20 years ago.

**Clinical findings:** the patient described lower limb edema with no other associated symptoms. Physical examination identified lower limb oedema and hypertension with a blood pressure of 162/82 mmHg. She presented with pure nephrotic syndrome (haematuria, hypertension, and renal failure). The 24-hour urine collection showed 6 g protein, the estimated glomerular filtration rate (EGFR) at 40 ml/min /1.73m^2^. She received anti-proteinuric treatment, her blood pressure was controlled on Irbesartan 300mg/day and Lercanidipine 20mg/day.

**Timeline of the current episode:** this lower limb edema had progressively appeared three weeks before hospitalization. From March 2021 to May 2021, hospitalized for edema of the lower limbs and nephrotic syndrome, with a renal biopsy diagnosing MN. During the same hospitalization, a pulmonary adenocarcinoma was discovered. In July 2021, oncology management: surgical resection of the tumor. December 2021, persistence of nephrotic syndrome despite tumor remission, Anti-PLA2R positive. February 2022, start of immunosuppressive therapy. September 2022, normalization of renal function and reduction of proteinuria.

**Figure 1:**
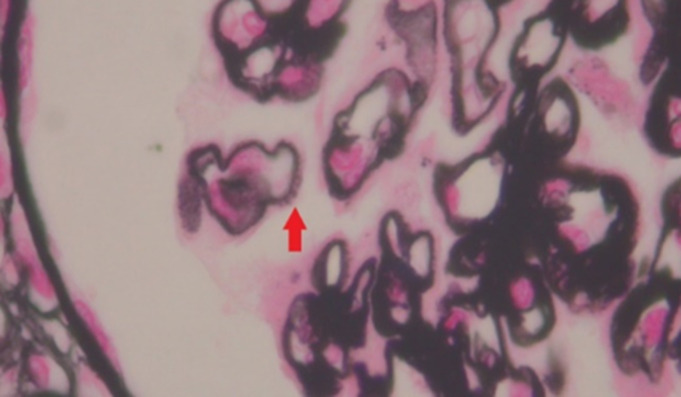
histology of renal biopsy, light microscopy (silver stain coloration): diffuse thickening of the glomerular capillary wall with the presence of spikes

**Figure 2:**
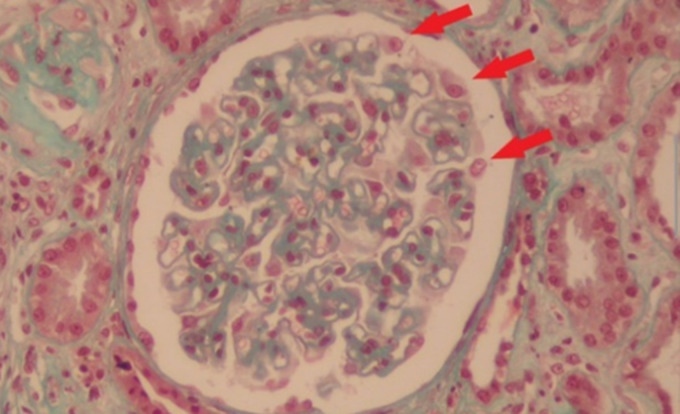
histology of renal biopsy, light microscopy (Masson trichrome coloration): turgid and detached podocytes

**Diagnostic assessment:** renal biopsy showed diffuse thickening of the glomerular capillary wall in light microscopy, spike formation on silver stain, and granular IgG deposits along the capillary wall by IF (Figure 1, Figure 2). Based on the findings on light, electron, and immunofluorescence microscopy, the diagnosis of membranous nephropathy was made. The findings prompted the following laboratory workup to determine etiology. The antinuclear antibody test, serum C3 and C4 complement levels, and hepatitis B and C and HIV serologies were within reference ranges. Anti-TSH receptor was positive, anti-TPO was negative, with normal thyroid function tests. In the absence of exophthalmos and goitre, the advice of the endocrinologists was to recheck the Anti-TSH after 6 months. Standard chest radiographs were ordered. They showed a suspicious image of the left lung, and it was a lung nodule of the left upper lobe with a pleuro-parietal attachment point with poly-lobed contours at the chest CT scan. The diagnosis of invasive adenocarcinoma of the lung, classified T1BN0M0, was retained.

**Therapeutic intervention:** she had a complete resection of the tumor. The diagnosis of secondary MN was considered. The patient was rechecked 6 months later, and oncological follow-up did not show any recurrence of the tumor but persistence of nephrotic syndrome. Anti-PLA2R antibody titre was not measured at initial patient presentation. The assay was performed 6 months after the onset of the disease. Antibodies came back positive (214.3 UR/ml), making the diagnosis of primary MN, though they were checked late. Based on this result, we initiated corticosteroid therapy with cyclophosphamide according to the Ponticelli protocol and continued the maximum dose of angiotensin receptor blocker (ARBs).

**Follow-up and outcomes:** within 6 months of therapy, she responded very well with improvement in proteinuria from 6 g to only 1.7 g. His renal function decreased to 63 μmol/l with the disappearance of the oedemas. She remained on immunosuppressive treatment (2g of Mycophenolate Mofetil) and 10mg of corticosteroid. Anti-PLA2R antibodies have been checked and returned negative. It helped in confirming the diagnosis of primary MN. Three years later, she has been doing very well with <2 g of proteinuria, normal renal function, and no recurrence of the lung tumor. According to this case report, the question that arose: is it a primary MN incidentally associated with the lung tumor or a secondary MN, but with positive Anti-PLA2R? The initial hypothesis appears to be the most plausible.

**Patient's perspective:** “Currently, I feel much better. Before, I was very bothered by edema. In addition, I had been diagnosed with cancer, and I thought that my kidney disease would be cured after treatment for my tumor. When things got worse after my tumor was being treated, I was very scared. But thank God, my doctor reassured me, and after taking the tablets, everything disappeared, and I had no more edema. I was very lucky; I had cancer and kidney disease, but I came out of it well.”

**Informed consent:** written informed consent was obtained from the patient for the publication of anonymized data in this article.

## Discussion

Membranous nephropathy is the most common cause of nephrotic syndrome in the adult population. Approximately 80% of MN cases are primary; the remainder are secondary to conditions such as malignancy, infectious disease, or an autoimmune disorder [1]. Primary and secondary MN can easily be distinguished by clinical, laboratory, and histologic features. The presence of anti-PLA2R autoantibodies allows clinicians to quickly and accurately diagnose primary MN. Sometimes, distinguishing the two forms can be a diagnostic challenge, and subsequently a therapeutic challenge, especially in the presence of an evident etiology but with positive anti-PLA2R autoantibodies; it is generally the course of evolution and the response to treatment that clarifies the situation. In the case of our patient, we have two hypotheses.

### Hypothesis 1: it is a secondary MN but with positives anti PLA2R

In our case, this hypothesis had powerful epidemiological, etiological, and chronological arguments. In fact, our 68-year-old patient had lung cancer, and her tumor was diagnosed during etiological assessment of the nephropathy. According to the literature, the prevalence of cancer in MN varies across studies; a meta-analysis involving 6 individual study populations, including 785 patients, concluded that the prevalence of cancer ranged between 4.8 and 20.49% with an overall meta-analytical prevalence of 10% [2]. The most common cancers related to MN were lung, prostate, and hematologic, respectively 25,15 and 14% of cases. The average age was 66 years. Regarding the timing of cancer diagnosis, Leeaphorn *et al*. reported that only 20% of MN patients had the diagnosis of cancer before the diagnosis of MN, and it was diagnosed at the time or following the diagnosis of MN in 80% of cases [2]. In our case, the patient did not have any symptom and the discovery of cancer was incidental during the screening process of the nephropathy.

The pathogenic mechanism of MN occurrence in malignancy is not yet elucidated. Beck proposed that cancer patients have increased levels of CICs (immune response mounted against tumor-expressed antigens). Alternatively, the tumor may present an otherwise immunologically privileged antigen, or by molecular mimicry, provoke a humoral response against a normal host protein. It is also conceivable that an extrinsic process is responsible for both malignancy and MN [3]. Some histologic particularities may be predictive of malignancy-associated MN; glomerular IgG4 deposition was identified predominantly in primary MN, whereas predominant depositions of IgG1/IgG2/IgG3 were often seen in secondary MN, including malignancy-related MN. The absence of glomerular IgG4 deposition was an independent predictor for the development of malignancy. In our case, we did not do IgG typing. Another histologic particularity, the presence of > 8 inflammatory cells per glomeruli on kidney biopsy, is strongly associated with MN-related cancer. It was observed by Lefaucheur *et al*. in a study on cancer-related membranous glomerulonephritis [4].

Regarding the serological profile, the identification of autoantibodies largely associated with primary MN began with the discovery of anti-PLA2R [5]. It has been proven that they are specific in 80% of cases in Runco´s updates [6]; in other series, it varied (0-60%). However, their presence does not exclude secondary MN. A study including 184 secondary MN evaluated the prevalence of anti-PLA2R antibody, which was positive in 60% of patients with hepatitis B, 64% of patients with hepatitis C, 75% in patients with sarcoidosis, and 70% of patients with malignancies [7]. Another antibody could be more interesting for diagnosing malignancy-associated membranous nephropathy. A recent study by Caza *et al*. confirmed that NELL1 (nerve epidermal growth factor-like 1) could be the first candidate antigen in malignancy-associated membranous nephropathy. Caza *et al*. in a group of 91 patients with secondary MN, reported the presence of NELL1 antigen and IgG1 deposits in 93.4% of cases of secondary MN to cancer [8].

### Hypothesis 2: it is an idiopathic MN incidentally associated with lung cancer

This hypothesis also has arguments. In fact, in our case, in addition to the positive anti-PLA2R, there was no remission after tumor resection. The nephrotic syndrome of our patient persisted after resection, and the clinical response was only obtained after the introduction of immunosuppressive therapy.

Case series have reported PLA2R positivity in patients with idiopathic MN associated with solid organ malignancy. A Chinese study explored the prevalence of auto-antibodies against PLA2R in patients with idiopathic MN and included secondary MN cases to confirm the specificity of anti-PLA2R for idiopathic MN [9]. Eighty-one per cent of idiopathic MN were positive for anti-PLA2R antibodies. Ten patients were considered secondary MN; three of them had a tumor and positive anti-PLA2R. During their follow-up, they had persistent or relapse of proteinuria despite resection of the tumor, which was the case of our patient, and that was in favor of the coincident association of cancer with idiopathic MN. In patients without anti-PLA2R autoantibodies and presenting MN-associated organ malignancy, complete remission of the proteinuria was seen in two patients after tumor resection; three patients died of cancer; the remaining two patients had persistent proteinuria without tumor resection. Baker *et al*. reported a case of biopsy-proven MN in a patient with positive serologic anti-PLA2R autoantibodies and 2 simultaneous active malignancies [10], who was treated by chemotherapy and radiotherapy with partial renal response.

## Conclusion

We presented a rare case of biopsy-proven MN with high serum anti-PLA2R antibodies associated with lung cancer. Several arguments argue in favor of the idiopathic nature of nephropathy, especially the good evolution under immunosuppressive treatment. We emphasize that, in light of the lack of improvement of nephrotic syndrome after treatment of the underlying cause of MN, it is important to reconsider the diagnosis and to treat the nephropathy as primary. However, we insist on the importance of cancer screening in patients with newly diagnosed MN, since the use of immunosuppressive medications can have a deleterious effect on tumor growth.
